# The impact of gut microbiota on leukemia and prospects for novel therapies

**DOI:** 10.1016/j.imj.2026.100239

**Published:** 2026-02-10

**Authors:** Saba Jalalifar, Bahar Bajelan, Reihane Mohammadi, Roya Ghafoury, Zahra Kalhori, Kamran Pooshang-Bagheri, Reza Nekouian, Mohammad Faranoush

**Affiliations:** a Pediatric Growth and Development Research Center, Institute of Endocrinology and Metabolism, Iran University of Medical Sciences, Tehran 1593716615, Iran; b Mehresoheila Cancer Charity, Alborz 3139954344, Iran; c School of Medicine, Alborz University of Medical Sciences, Karaj 3149779453, Iran; d Endocrine Research Center, Institute of Endocrinology and Metabolism, Iran University of Medical Sciences, Tehran 1593716615, Iran; e Department of Medical Library and Information Science, School of Health Management and Information Science, Iran University of Medical Science, Tehran 1449614535, Iran; f Venom and Biotherapeutics Molecules Lab, Medical Biotechnology Department, Biotechnology Research Center, Pasteur Institute of Iran, Tehran, 1316943551, Iran

**Keywords:** Microbiome, Leukemia, Gut microbiota, Microbiome-targeted treatment, Probiotic, Fecal microbiota transplantation

## Abstract

The Human Microbiome Project has underscored the pivotal role of the gut microbiome in human health, revealing its potential influence on leukemia development, progression, and treatment response. This review summarizes evidence on microbiome-targeted therapies such as probiotics, fecal microbiota transplantation, antimicrobial peptides, and nanoparticles. These approaches may improve leukemia treatment outcomes through immune and metabolic modulation and reduced toxicity. Although emerging data suggest beneficial effects, most findings remain correlative and limited by small, heterogeneous studies. Further mechanistic and clinical research is required to clarify causal pathways, standardize interventions, and evaluate long-term safety. Personalized microbiome-based strategies that integrate molecular and immunologic profiling may ultimately refine leukemia management and improve survival.

## 1 Introduction

The human microbiome is a collection of dynamic microbial communities including bacteria, archaea, fungi, and viruses, that inhabit multiple body sites and contribute to host metabolism, immunity, and tissue homeostasis.^1^ Disruption of this ecosystem, termed dysbiosis, is characterized by reduced microbial diversity, loss of health-associated taxa, and expansion of potentially pathogenic microbes, with contributing factors such as antibiotics, diet, and host genetics. Dysbiosis has been associated with systemic inflammation, immune imbalance, and increased disease susceptibility, linking the microbiome to both metabolic and neoplastic disorders.^1^, ^2^, ^3^ Advances in metagenomics and metabolomics have expanded understanding of the gut microbiome's composition, function, and host interactions, revealing opportunities for therapeutic modulation. These insights have driven the development of microbiome-based interventions, such as dietary modification, probiotics, and fecal microbiota transplantation (FMT), aimed at restoring microbial equilibrium and supporting immune and metabolic homeostasis. Because microbial imbalance contributes to chronic inflammation and impaired host defenses, targeting dysbiosis represents a promising complementary approach to disease prevention and treatment.^4^, ^5^, ^6^, ^7^, ^8^ Hematologic malignancies such as leukemia are characterized by uncontrolled proliferation driven by genetic and epigenetic alterations and remain a major cause of mortality. Recognizing cancer as a heterogeneous group of diseases rather than a single entity underscores the need for diverse investigative approaches to understand its molecular complexity and therapeutic vulnerabilities.^9^^,^^10^ Leukemia comprises biologically diverse hematologic malignancies including acute lymphoblastic leukemia (ALL), acute myeloid leukemia (AML), chronic lymphocytic leukemia (CLL), and chronic myeloid leukemia (CML) that differ in pathogenesis and treatment response. Mounting evidence connects gut microbiome composition with leukemia risk, treatment tolerance, and clinical outcomes, positioning the microbiome as a key element in disease biology and supportive care.^11^, ^12^, ^13^ In leukemia, intensive chemotherapy, antibiotics, and immunosuppressive regimens can profoundly disturb gut microbial diversity, while pre-existing or treatment-shaped microbiota may, in turn, influence therapy efficacy, toxicity, and infection risk. Understanding this bidirectional relationship provides a rationale for microbiome-based interventions aimed at restoring microbial balance and improving patient outcomes.^14^^,^^15^ This review synthesizes evidence on the interplay between the gut microbiota and leukemia, emphasizing mechanistic insights and the therapeutic potential of microbiome-modulating strategies such as probiotics, prebiotics, and FMT. It also outlines current limitations and future directions for integrating microbiome research into leukemia management and personalized therapy.

## 2 The gut microbiome in leukemia: Mechanisms and clinical implications

### 2.1 Microbiome–leukemia interactions: Evidence and pathogenic links

Leukemia, a group of hematopoietic malignancies, has been linked to alterations in the gut microbiome, which profoundly influence host metabolism, immunity, and inflammation. Emerging evidence suggests that gut dysbiosis may contribute to leukemia initiation and progression by disrupting immune surveillance and promoting chronic inflammatory states. For example, decreased microbial diversity and enrichment of genera such as *Blautia* and *Lactococcus* have been observed in ALL, whereas *Rikenellaceae* and *Anaerostipes* have been associated with AML.^16^^,^^17^ Although these associations do not establish direct causality, they highlight the potential role of microbial imbalance in shaping leukemogenic microenvironments and influencing disease susceptibility.

**Fig. 1:**
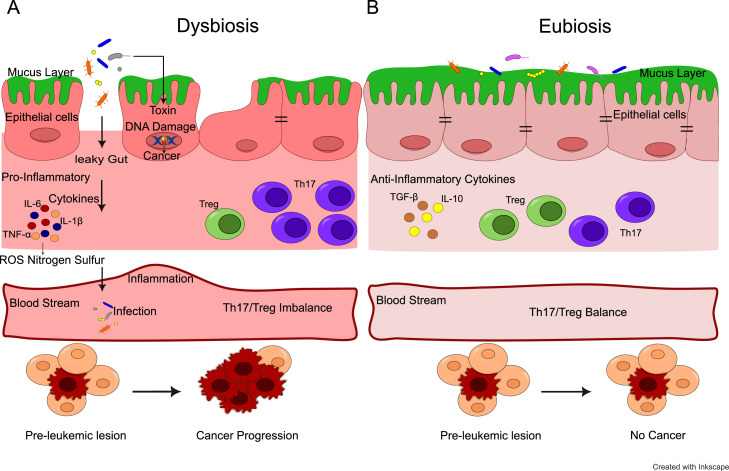
Mechanisms of the gut microbiota which could lead to leukemia. (A) Dysbiosis. An imbalance in gut bacteria will damage DNA, release some pro-inflammatory factors, facilitate the necrotic process by reactive oxygen species (ROS), imbalance in immune system ingredients, inflammation, and infections. (B) Eubiosis.

Mechanistically, dysbiosis can perturb immune and metabolic homeostasis. Altered microbial communities may drive pro-inflammatory cytokine production, such as interleukin-6 (IL-6), IL-17, and tumor necrosis factor-α (TNF-α), through activation of the nuclear factor-kappa B (NF-κB) and Janus kinase/signal transducers and activators of transcription (JAK/STAT) pathways, thereby fostering a microenvironment conducive to malignant transformation.^18^^,^^19^ In parallel, loss of short-chain fatty acid (SCFA) -producing bacteria such as *Faecalibacterium* and *Roseburia* can reduce concentrations of butyrate and propionate, metabolites known to regulate Treg differentiation, histone deacetylase (HDAC) activity, and epithelial integrity.^20^^,^^21^ The resulting immunometabolism imbalance may impair antitumor immunity and facilitate leukemic proliferation.^18^, ^19^, ^20^ Collectively, these immune and metabolic disturbances outline plausible pathways linking dysbiosis to leukemogenesis (Fig. 1).

Conversely, leukemia and its treatments exert reciprocal effects on gut microbial structure. Cytotoxic chemotherapy and prophylactic antibiotics commonly used in ALL and AML, can induce profound dysbiosis characterized by depletion of commensal taxa and expansion of opportunistic pathogens. This imbalance not only increases susceptibility to systemic infections but may also impair mucosal immunity and hematopoietic recovery.^19^^,^^21^^,^^22^ Such bidirectional interactions underscore the complexity of microbiome–host dynamics in leukemia pathophysiology.

These insights provide a biological rationale for microbiome-targeted interventions. Preliminary evidence indicates that probiotics, prebiotics, and dietary modulation may restore microbial diversity, enhance mucosal immunity, and reduce therapy-related complications. For instance, administration of *Lactobacillus rhamnosus GG* or *Bifidobacterium longum* has shown potential in mitigating chemotherapy-induced mucositis and improving immune function in hematologic malignancies.^16^^,^^19^^,^^21^ While data remain limited and largely preclinical, the growing understanding of immune and metabolic pathways linking the gut microbiome to leukemia supports exploration of these strategies as adjuncts to conventional therapy. Ongoing studies integrating metagenomics, metabolomics, and immune profiling are expected to clarify causal relationships and guide the development of personalized microbiome-based therapeutics for leukemia.

### 2.2 Leukemia- and therapy-induced alterations in the gut microbiome

Leukemia and its intensive treatments markedly remodel the intestinal microbiome. Chemotherapy, antibiotic prophylaxis, and hematopoietic stem-cell transplantation (HSCT) frequently induce dysbiosis characterized by diminished microbial diversity, depletion of *Firmicutes* and *Bacteroidetes*, and overgrowth of opportunistic taxa such as *Enterococcus* and *Enterobacteriaceae*. These compositional shifts correlate with mucosal barrier injury, immune suppression, and higher rates of bloodstream infection and graft-versus-host disease (GVHD).^23^, ^24^, ^25^

#### 2.2.1 Immune dysregulation

Chemotherapy-induced mucositis disrupts epithelial integrity, allowing microbial translocation and release of lipopolysaccharide and other microbial products into the bloodstream. These act as pathogen-associated molecular patterns that trigger Toll-like receptor (TLR) and NOD-like receptor signaling, driving IL-6, IL-1β, and TNF-α production via NF-κB activation. The resulting systemic inflammation not only worsens mucosal injury but can impair hematopoietic recovery and promote infection-related mortality.^26^, ^27^, ^28^ Antibiotic-driven loss of SCFA-producing bacteria (e.g., *Faecalibacterium, Roseburia*) further reduces butyrate levels, limiting epithelial regeneration and regulatory T-cell differentiation.^29^^,^^30^ Such immune-metabolic perturbations establish a vicious cycle of barrier dysfunction, cytokine dysregulation, and microbiota imbalance.

#### 2.2.2 Chronic inflammation, tissue damage, and disease progression

Persistent inflammation is a key mediator linking gut dysbiosis with leukemia progression and treatment-related complications. Persistent dysbiosis after chemotherapy increases intestinal permeability and systemic exposure to microbial products, sustaining NF-κB-driven cytokine production (IL-1β, IL-6, TNF-α). The resulting cytokine storm amplifies mucosal damage, delays epithelial regeneration, and promotes oxidative stress within the bone-marrow and intestinal microenvironments.^31^^,^^32^ Chronic activation of these pathways sustains systemic inflammation that can impair hematopoietic recovery and immune regulation, fostering conditions conducive to leukemic cell survival. Reduced levels of SCFA-producing genera (*Faecalibacterium, Roseburia*) further limit butyrate availability, weakening intestinal barrier repair and anti-inflammatory signaling through G-protein-coupled receptors and histone deacetylase inhibition. Mitochondrial dysfunction and epithelial apoptosis perpetuate barrier loss, completing a self-reinforcing cycle of inflammation and tissue injury.^33^ Collectively, these findings indicate that therapy-induced dysbiosis and inflammation act synergistically to exacerbate mucosal injury, systemic immune imbalance, and potentially leukemic persistence. Interventions aimed at preserving microbial diversity and restoring mucosal integrity such as prebiotic supplementation, targeted probiotics, or nutritional modulation, represent promising adjuncts for mitigating inflammation-driven complications in leukemia management.

#### 2.2.3 Leukemia-specific patterns

Recent research indicates that patients with ALL have a diminished diversity of gut microbes, which is linked to the advancement of the disease and its treatment outcomes. Children with acute ALL exhibit baseline enrichment of *Blautia* and depletion of *Bifidobacterium*, patterns that intensify after multi-agent chemotherapy.^16^^,^^17^ In AML, induction therapy rapidly diminishes microbial richness; the degree of loss in butyrate-producing taxa predicts febrile neutropenia and prolonged hospitalization.^21^^,^^34^ In CML, increased abundance of *Streptococcus* species and reduction of *Lactobacillus* have been linked to low-grade intestinal inflammation and altered cytokine signatures.^35^^,^^36^ CLL patients similarly display reduced α-diversity and enrichment of pro-inflammatory taxa that correlate with elevated C-reactive protein (CRP) and IL-6 levels.^22^^,^^37^ Although causal direction remains uncertain, these findings collectively indicate that leukemic state and therapy profoundly shape the microbial landscape.

#### 2.2.4 Clinical implications

Therapy-induced dysbiosis contributes to complications including infection, GVHD, and treatment resistance. Conversely, preservation or restoration of commensal anaerobes such as *Blautia* and *Bifidobacterium* has been associated with improved immune reconstitution and survival after HSCT.^38^^,^^39^ This understanding supports microbiome-conscious management strategies, judicious antibiotic use, nutritional modulation, and investigational probiotic or FMT approaches, to maintain microbial equilibrium during leukemia care.^40^ Integrating longitudinal microbiome monitoring into clinical protocols may ultimately enable personalized interventions that mitigate dysbiosis-related toxicity and enhance therapeutic efficacy.

### 2.3 Mechanistic insights: Immune and metabolic crosstalk

#### 2.3.1 Microbiome-induced changes in leukemia

The gut microbiome can influence leukemogenesis and disease progression through immune, metabolic, and genotoxic mechanisms that collectively shape the hematopoietic microenvironment. Microbial products such as lipopolysaccharides and peptidoglycan fragments engage TLR and NOD-like receptors on epithelial and immune cells, activating downstream NF-κB and signal transducer and activator of transcription 3 (STAT3) signaling cascades that drive production of IL-6, TNF-α, and other pro-inflammatory cytokines.^41^ Persistent activation of these pathways promotes oxidative stress and DNA damage in hematopoietic stem and progenitor cells, favoring clonal expansion and malignant transformation.

Beyond inflammation, dysbiosis alters the host metabolic milieu. Reduction of SCFA-producing bacteria (*Faecalibacterium, Roseburia, Anaerostipes*) decreases butyrate availability, weakening HDAC inhibition and regulatory T-cell induction, thereby weakening mechanisms that normally restrain aberrant proliferation.^41^^,^^42^ Loss of microbial control over bile-acid and tryptophan metabolism further amplifies leukemogenic signaling via farnesoid-X-receptor and aryl hydrocarbon receptor pathways.

The leukemia microenvironment itself may foster selective microbial colonization. Hypoxia, nutrient imbalance, and treatment-induced mucosal damage create niches for opportunistic taxa whose metabolites exacerbate immune dysregulation and impair antitumor surveillance.^42^, ^43^, ^44^, ^45^ These interactions illustrate how microbiome-derived immune and metabolic cues can modulate hematopoietic homeostasis and therapeutic response.

Collectively, microbiome-induced inflammatory and metabolic perturbations establish systemic conditions that can accelerate leukemic evolution and influence treatment efficacy. Deciphering these mechanisms supports the development of microbiome-modulating interventions aimed at restoring immune equilibrium, protecting genomic integrity, and enhancing outcomes in leukemia patients.

#### 2.3.2 Leukemia- and cancer-induced changes in the gut microbiome

Malignancy itself, and its associated metabolic and immune alterations, can reshape the gut microbial ecosystem. In leukemia, systemic inflammation, immune suppression, and therapy-induced mucosal injury create selective pressures that favor opportunistic bacterial expansion and loss of commensal diversity. Elevated circulating cytokines such as IL-6 and TNF-α alter intestinal permeability and nutrient flux, disrupting anaerobic niches and enabling colonization by facultative pathogens.^41^^,^^46^ The leukemic state is often accompanied by catabolic metabolism and oxidative stress that modify bile-acid and amino-acid availability. These metabolic shifts, in turn, influence microbial community structure; for example, altered bile-acid composition can suppress *Bacteroides* and promote *Enterococcus* proliferation, while increased reactive oxygen species reduce obligate anaerobes essential for butyrate production.^41^^,^^47^ Reduced concentrations of short-chain fatty acids weaken epithelial barrier function and regulatory T-cell signaling, amplifying dysbiosis and systemic inflammation.

Cancer-related dysbiosis also exhibits systemic consequences. Microbial metabolites and inflammatory mediators generated in the gut can circulate to distant sites, affecting hepatic metabolism and hematopoietic niches.^48^^,^^49^ This systemic crosstalk contributes to chronic immune activation and may influence bone-marrow microenvironments that support leukemic cell survival. Understanding how leukemia-driven metabolic and immune disturbances remodel the gut microbiome is crucial for designing therapeutic interventions. Strategies that preserve commensal stability during intensive treatment, through dietary modulation, selective antibiotic stewardship, or microbiota restoration, may help break this feedback loop between malignancy, inflammation, and dysbiosis, ultimately improving clinical outcomes.^50^^,^^51^

### 2.4 Microbiota and treatment response

Leukemia therapy profoundly alters gut microbial composition; conversely, these microbial changes can in turn influence treatment efficacy and toxicity. The gut microbiome is increasingly recognized as a critical determinant of therapeutic outcomes in leukemia, shaping both efficacy and toxicity of treatment through complex immune-metabolic and barrier-mediated pathways. In patients undergoing induction chemotherapy, baseline low α-diversity of gut flora has been associated with higher rates of febrile neutropenia, bloodstream infection and delayed neutrophil recovery.^16^^,^^35^ A large observational study in acute leukemia found that diminished microbial richness prior to treatment correlated with poorer overall survival and higher complication rates.^52^

Chemotherapy and antibiotic use exert bidirectional effects: cytotoxic regimens and mucosal injury trigger microbial dysbiosis characterized by loss of SCFA-producing commensals (e.g., *Faecalibacterium, Roseburia*) and relative expansion of opportunistic taxa such as *Enterococcus* and *Enterobacteriaceae.*^53^^,^^54^ Resultant SCFA depletion weakens epithelial integrity and increases permeability, facilitating translocation of microbial products and downstream inflammatory signaling.^55^

In the setting of allogeneic HSCT, peri-transplant microbial profiles have emerged as strong prognostic indicators. Higher stool microbial diversity at neutrophil engraftment has been independently associated with lower risk of acute GVHD, reduced transplant-related mortality and improved overall survival.^56^ Specific taxa such as *Blautia* and *Bifidobacterium* (butyrate producers) corresponded with better outcomes, whereas domination by *Enterococcus* predicted worse survival. Mechanistically, preservation of commensal anaerobes supports regulatory T-cell recovery, limits alloreactive T-cell expansion and mitigates inflammatory cytokine release.^57^

Emerging data also implicate the microbiome in immunotherapy responsiveness. Although direct leukemia-specific studies remain limited, extrapolated evidence from solid-tumour settings shows that broad-spectrum antibiotic usage prior to immune checkpoint therapy correlates with reduced T-cell activation and inferior response rates.^58^ In leukemia contexts such as chimeric antigen receptor T-cell therapy (CAR-T cell), altered microbiome composition has been associated with increased cytokine-release syndrome severity and poorer remission durability. Pharmacomicrobiomic mechanisms further underscore the microbiome's role: bacterial β-glucuronidases and azoreductases can modify chemotherapy prodrugs and affect their bioavailability or toxicity; dysbiotic microbiota may reduce drug activation and increase off-target damage.^59^^,^^60^

Given this convergence of evidence, integrating microbiome profiling into leukemia therapeutic protocols offers a promising strategy: monitoring microbial composition before, during and after treatment may identify high-risk patients, guide antibiotic stewardship, and support adjunctive microbiota-modulating interventions (e.g., diet, prebiotics, selective probiotics) aimed at maintaining microbial diversity and improving treatment outcomes.^61^

## 3 Emerging approaches in leukemia treatment

**Fig. 2:**
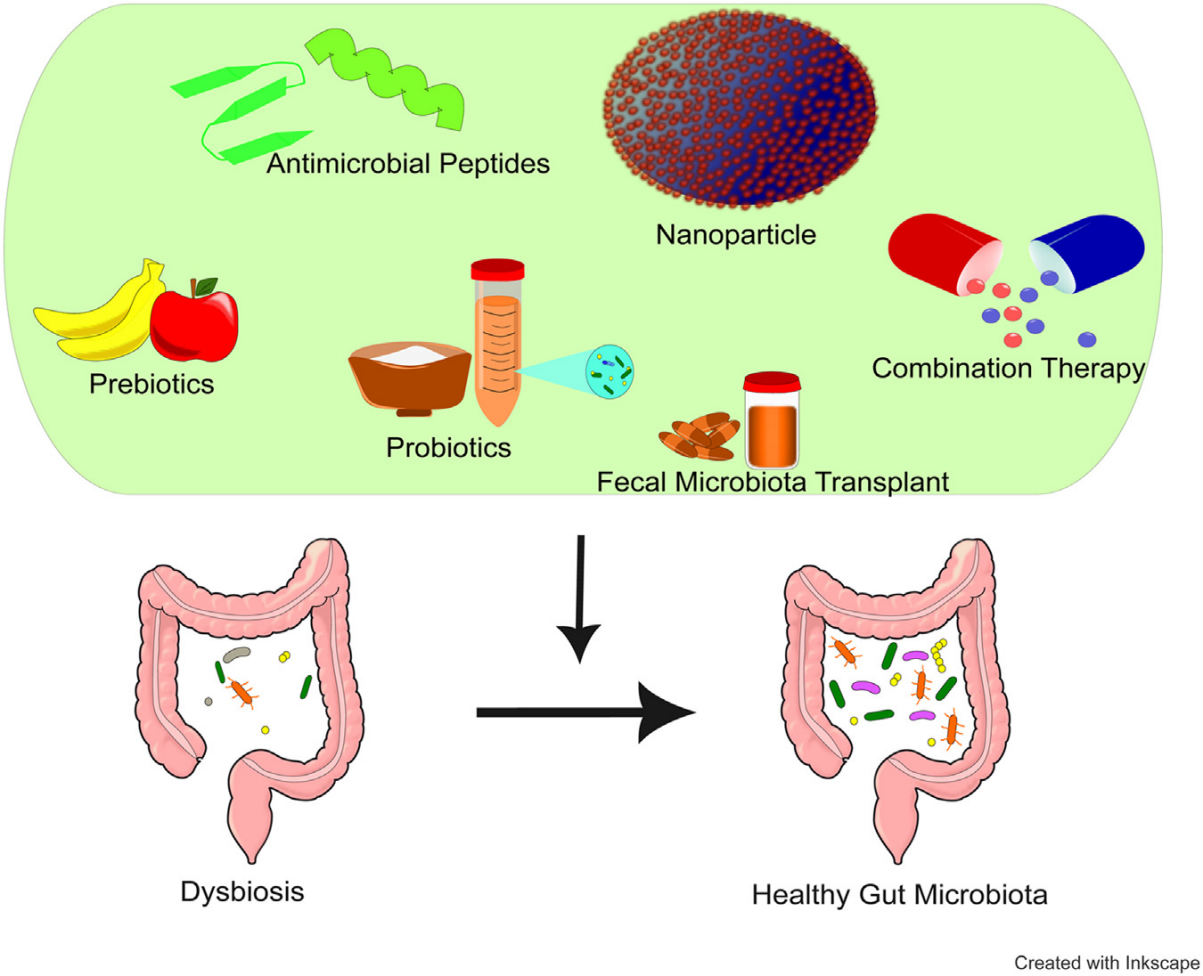
Role of emerging therapeutic approaches in leukemia treatment. Probiotics, fecal microbiota transplantation (FMT), peptides, antimicrobial peptides (AMPs), nanoparticles, and combination therapies contribute to establishing a more stable gut environment, potentially mitigating leukemia-related complications by restoring intestinal balance and supporting overall treatment efficacy.

The gut microbiome influences leukemia progression through intertwined immune, metabolic, and inflammatory pathways. These same mechanisms provide a biological rationale for therapeutic strategies that modulate host–microbe interactions. Current interventions either restore microbial balance disrupted by leukemia and its treatment (e.g., probiotics, FMT) or harness microbiota-derived molecules such as antimicrobial peptides (AMPs), bioactive peptides, and nanoparticles, to enhance anti-leukemic efficacy.^19^ Restoration of a healthy microbial ecosystem may reduce inflammatory activation, improve intestinal barrier function, and optimize systemic immune responses, all of which can influence treatment effectiveness. FMT, for instance, has shown promise in restoring microbial diversity in AML patients after chemotherapy or antibiotic therapy, improving dysbiosis and potentially preventing infection-related morbidity.^19^^,^^62^ Emerging studies indicate that certain lactic acid bacteria possess antitumor activity through production of SCFAs and modulation of immune checkpoints.^19^ Engineered or metabolically modified probiotics may further enhance immunotherapy outcomes by reshaping the metabolic profile of the tumor microenvironment. In addition, combined prebiotic–probiotic (synbiotic) regimens such as those using inulin, represent a complementary approach to reinforce beneficial microbial populations and immune resilience, though larger controlled studies are needed to confirm their efficacy in leukemia (Fig. 2, Table 1).^19^

**Table 1:** Current approaches to leukemia treatment.

Items	Probiotics	FMT	Nanoparticles	Peptide
**Mechanisms of action**	1. Immune modulation 2. Gut barrier integrity 3. Reduction of treatment side effects 4. Enhancing treatment efficacy^16^^,^^63^^,^^64^	Enhance gut microbiota balance^19^^,^^62^^,^^65^	1. Targeted drug delivery 2. Improved drug solubility and bioavailability 3. Overcoming drug resistance^66^^,^^67^	1. Therapeutic peptides (for example L- and D-peptides): a) Promotes differentiation in human AML cells b) Facilitates maturation c) Increases the expression of CD11b and CD14^68^ 2. Bioactive peptides: a) Inhibit the proliferation of AML cells b) Encourage apoptosis c) Associated with downregulation of specific mRNA modifications (m^6^A demethylation)^69^ 3. Immunopeptidome In CLL, a T-cell-based immunotherapies targeting these peptides could improve patient outcomes^70^^,^^71^ 4. AMPs a) Induction of cell death b) Synergistic effects with chemotherapy^72^^,^^73^
**Clinical trials**	1. Diarrhea**/** *Lactobacillus plantarum* strain 299v, *Lactobacillus acidophilus*, and *Bifidobacterium lactis*^74^ 2. Chemotherapy-induced gastrointestinal side effects**/***Lactobacillus rhamnosus*^75^^,^^76^ 3. Oral mucositis in pediatric leukemia patients^77^	**—**	**—**	**—**
**Potential benefits**	1. Alleviate gastrointestinal side effects 2. Enhance the overall quality of life for patients 3. Reducing the risk of infections among immunosuppressed patients^75^^,^^78^	1. Restoration of gut microbiota 2. Enhanced immunotherapy effectiveness 3. Mitigation of treatment-related complications 4. Safety profile^62^^,^^79^	Improve the effectiveness of chemotherapy^19^	More effective treatment strategies with focusing on particular mechanisms^19^
**Limitations**	1. Safety concerns: systemic infections and bacteremia 2. Variable efficacy 3. Lack of comprehensive evidence^78^^,^^80^	1. Limited impact on infection rates 2. Unclear mechanisms of action 3. Variability in donor microbiota 4. Need for further research^65^^,^^79^^,^^81^	1. Toxicity risks 2. Regulatory hurdles^19^	1. Complexity of mechanisms 2. Limited clinical trials^19^
**Outcome**	May help: 1. Alleviate side effects 2. Enhance recovery times^75^^,^^78^	Minimizing complications linked to chemotherapy^62^^,^^79^	1. Nanocarriers for chemotherapy 2. Diagnostic purposes 3. Combination therapies 4. Future directions^19^	1. Therapeutic peptides (for example L- and D-peptides): Differentiation therapy for AML patients^68^ 2. Bioactive peptides: improved overall survival rates in patients with AML^53^ 3. Analyzing the immunopeptidome may enhancing prognosis through personalized treatment strategies^53^ 4. AMPs: By their dual functionality, decreases the risk of resistance development and use against resistant leukemia forms^72^^,^^73^

*Abbreviations*: FMT, fecal microbiota transplantation; AML, acute myeloid leukemia; CLL, chronic lymphocytic leukemia; AMP, antimicrobial peptides.

### 3.1 Probiotics

Probiotics may improve outcomes in leukemia by modulating gut microbial composition and immune function.^16^^,^^82^ By modulating microbial metabolites such as SCFAs, probiotics can regulate hematopoietic and immune signaling pathways, including IL-10 and Treg activation, that are disrupted in leukemia.

Probiotics can strengthen immune responses by stimulating immunoglobulin production and regulating inflammation. They also help maintain intestinal barrier stability, as barrier disruption can trigger systemic inflammation and worsen leukemia progression.^63^^,^^64^ trains that produce butyrate, such as *Faecalibacterium*, may aid barrier repair, decrease inflammation, and improve overall health in individuals with leukemia.^83^ By reinforcing epithelial junctions and reducing circulating endotoxins, these microbes may limit chemotherapy-induced mucositis and secondary bacteremia. Incorporating probiotics during chemotherapy may alleviate gastrointestinal symptoms such as nausea and diarrhea and lower infection risk in immunocompromised patients.^84^^,^^85^ In addition, probiotic restoration of microbial diversity may reduce dominance of opportunistic pathogens such as *Enterococcus*, which are commonly linked to post-chemotherapy infections.

Probiotics may also enhance chemotherapy efficacy by modulating metabolic pathways related to drug metabolism; for instance, specific *Lactobacillus* or *Bifidobacterium* strains have been reported to potentiate the cytotoxic activity of doxorubicin.^86^^,^^87^ Research suggests that leukemia patients exhibit significant structural imbalances in their gut microbiota compared to healthy individuals. These dysbiotic patterns have been associated with poorer treatment outcomes and an increased risk of infections during therapy.^88^ Early clinical studies in pediatric leukemia suggest that probiotics can mitigate treatment-related side effects and accelerate recovery.^76^ However, most studies remain small, heterogeneous, and short-term; large randomized controlled trials are required to confirm strain-specific efficacy and long-term safety in immunocompromised patients.

### 3.2 Fecal microbiota transplantation

FMT represents a novel therapeutic strategy that has garnered attention for its potential advantages in individuals diagnosed with AML.^89^ The outcomes for leukemia patients, especially those undergoing rigorous chemotherapy, can be notably influenced by dysbiosis. This condition frequently arises due to the antibiotic treatments administered to control infections associated with the disease. By re-introducing diverse microbial communities, FMT helps restore gut eubiosis and immune balance disrupted by chemotherapy and antibiotics, partly through recolonization with SCFA-producing genera such as *Faecalibacterium* and *Blautia* that support mucosal repair and reduce inflammation.

Recent investigations indicate that FMT is generally safe for AML patients and can enhance gut-microbiota balance. One study showed that FMT restored microbial diversity after antibiotic therapy but did not significantly reduce infection rates compared with placebo.^62^^,^^65^ Microbial restoration may nevertheless strengthen intestinal-barrier integrity and limit overgrowth of opportunistic pathogens such as *Enterococcus*, thereby reducing endotoxin-driven inflammation. Although infection rates were not significantly reduced, one trial reported fewer events in the FMT group (0.93 vs. 1.25 per 100 patient-days), suggesting a modest but inconclusive advantage.^62^^,^^65^ Restoration of gut microbiota through FMT may improve treatment outcomes by reducing chemotherapy-related complications. Preliminary data suggest that a healthier microbiome could enhance therapeutic response and prognosis, although larger studies are needed for confirmation.^16^^,^^90^

Although early findings are encouraging, existing evidence is limited by small, heterogeneous cohorts. Randomized controlled trials are needed to confirm efficacy and establish safe protocols for immunocompromised patients. Ongoing research aims to optimize FMT procedures and evaluate combination approaches with immunotherapy to enhance antitumor effects.^79^^,^^90^ Larger multicenter trials are warranted to determine the effectiveness of FMT in mitigating dysbiosis-related complications in leukemia.^65^^,^^91^

### 3.3 Nanoparticles

Nanoparticles (NPs) are emerging as valuable tools for improving leukemia prognosis through enhanced diagnostics and targeted therapy.^92^ Growing evidence indicates that NP behavior and biocompatibility can be modulated by the host microbiome, as microbial metabolites and enzymes may alter surface chemistry and drug-release kinetics.

NPs can deliver chemotherapeutic agents directly to leukemia cells, reducing injury to healthy tissue. Such targeted delivery minimizes systemic toxicity and improves therapeutic efficacy. This can be achieved through passive targeting, exploiting tumor-vessel permeability, or active targeting, in which NPs are functionalized with ligands that bind specific cancer-cell receptors.^66^^,^^93^ NPs also improve solubility and bioavailability of anti-leukemic drugs, enabling effective dosing with fewer adverse effects—an advantage for agents that are poorly water-soluble or require high doses.^67^^,^^94^ Drug resistance remains a major limitation in leukemia therapy. Nanoparticle formulations can mitigate this challenge by co-delivering multiple agents or enhancing intracellular uptake of existing drugs.^92^^,^^95^ Experimental designs with microbiota-responsive coatings enable drug release in response to bacterial enzymes or pH shifts characteristic of dysbiosis, directly linking nanomedicine to microbiome-targeted therapy. NPs can also encapsulate chemotherapeutics for controlled, site-specific release, a strategy that has shown promise in improving treatment efficacy for ALL and other leukemia types.^66^^,^^67^

NPs are also being developed for diagnostic applications, including imaging and biomarker detection. They enhance the sensitivity and specificity of leukemia diagnostics, enabling earlier detection and individualized treatment planning.^95^^,^^96^ Current studies explore combining NPs with immunotherapy and gene therapy; for example, iron-oxide nanoparticles serve dual roles as MRI contrast agents and targeted drug-delivery systems.^92^^,^^96^ Despite encouraging progress, most NP research remains preclinical, and long-term effects on immune regulation and the gut microbiome are not well defined. Overcoming challenges related to biocompatibility, off-target toxicity, and regulatory approval will be crucial for clinical translation.^93^^,^^94^ Continued advances in nanotechnology may yield more precise and effective therapies, ultimately improving outcomes for patients with leukemia.

### 3.4 Peptides

Recent studies suggest that peptides may improve outcomes in leukemia, particularly in AML and CLL. Certain therapeutic peptides mimic or modulate host–microbe signaling molecules, overlapping with microbiota-driven immune pathways such as TLR2 activation and cytokine regulation.^53^

A novel D-peptide, pep-P6, was shown to promote differentiation in human AML cells, increasing the expression of markers such as CD11b and CD14.^68^ In xenograft models, pep-P6 slowed disease progression, supporting its potential as a differentiation therapy for AML. Its activity occurs primarily through TLR2 pathway activation, leading to changes in cell morphology and function.^97^ Both L- and D-forms were active, although D-pep-P6 was more effective in suppressing tumor growth.^68^ Other bioactive peptides have been shown to inhibit AML-cell proliferation and induce apoptosis, partly through downregulation of mRNA modifications such as m⁶A demethylation, correlating with improved overall survival.^69^ These findings highlight peptides as promising therapeutic candidates against AML. However, most evidence remains preclinical, and interactions between peptide therapy and the gut microbiome are largely unexplored; further studies are needed to evaluate their stability, safety, and immunomodulatory effects *in vivo*.

### 3.5 Immunopeptidome diversity in CLL

In CLL, greater diversity of human leukocyte antigens (HLA) -presented peptides—the immunopeptidome—correlates with a more favorable disease course. Patients with broader immunopeptidomic profiles experience fewer relapses and longer progression-free survival.^70^^,^^98^ These findings suggest that T-cell-based therapies targeting such peptides may improve outcomes. Immunopeptidome profiling could also help identify patients likely to respond to immunotherapies, enabling personalized treatment strategies.^70^^,^^71^ Collectively, these results illustrate how host–microbe interactions and antigen presentation intersect within the tumor immune microenvironment, supporting therapeutic approaches that leverage microbial- or host-derived peptides to enhance antitumor immunity.

AMPs are emerging as innovative agents for leukemia therapy due to mechanisms distinct from conventional chemotherapy. Specific AMPs such as the PFR peptide, derived from lactoferrin, exhibit potent antitumor effects on leukemia cells. As many AMPs are naturally produced by commensal bacteria or epithelial cells in response to microbial cues, they form a functional bridge between the microbiome and innate immunity, potentially restoring antimicrobial and antitumor balance disrupted during leukemia treatment. The PFR peptide inhibits proliferation of leukemia cell lines including HL-60 and melittin (MEL) by inducing necrotic rather than apoptotic cell death, characterized by membrane disruption and increased intracellular calcium, without chromatin condensation or caspase activation.^72^^,^^73^ The necrotic process is facilitated by reactive oxygen species and mitochondrial dysfunction, which play crucial roles in the peptide's cytotoxic properties.^73^ The PFR peptide also shows synergy with standard chemotherapeutics such as cytosine arabinoside.^73^ This combination enhances necrotic effects on leukemia cells and markedly suppresses tumor growth *in vivo* without increasing toxicity to healthy tissue.^99^ These results suggest that AMPs may augment existing leukemia therapies by improving efficacy while minimizing adverse effects.

The dual functionality of AMPs, targeting both cancer cells and pathogens, positions them as innovative therapeutic agents in cancer treatment. Their ability to selectively disrupt cancer cell membranes while preserving healthy cells reduces the risk of resistance development, a persistent challenge in conventional chemotherapy.^100^^,^^101^ Moreover, AMPs can be engineered for improved stability and specificity, offering potential in refractory forms of leukemia.^100^ However, most data remain preclinical, and further studies are needed to evaluate AMP stability, immunogenicity, and microbiome interactions in clinical settings.

## 4 Benefits and limitations of emerging approaches

Recent advances in leukemia management, particularly in ALL, have introduced several innovative strategies, including probiotics, peptides, FMT, nanoparticles, and combination therapies. These modalities aim to restore gut-microbiota balance, strengthen immune regulation, and reduce complications when integrated with conventional treatments such as chemotherapy and hematopoietic stem-cell transplantation. Despite their promise, each approach presents unique challenges that require further investigation and optimization. Well-designed, standardized clinical trials are needed to confirm efficacy and ensure safety, especially in immunocompromised patients.

### 4.1 Probiotics

Research has investigated the role of probiotics in reestablishing gut microbiota that may be disrupted by chemotherapy, which could alleviate gastrointestinal side effects and enhance the overall quality of life for patients undergoing treatment.^75^^,^^78^ These effects are thought to result from restoring microbial diversity, increasing SCFA production, and improving gut-barrier integrity disrupted by chemotherapy. Additionally, probiotics might play a role in reducing the risk of infections among patients with weakened immune systems.^78^^,^^102^

The use of probiotics in patients with compromised immunity can lead to serious complications, including systemic infections and bacteremia. There have been documented instances where patients developed severe infections associated with specific probiotic strains.^78^^,^^80^ The success of probiotics can differ widely among patients due to variations in individual microbiota and overall health. While some research suggests that probiotics can lessen gastrointestinal symptoms, they do not consistently lead to improved health outcomes for every patient.^84^^,^^103^ Despite encouraging early data, evidence remains limited to small heterogeneous studies, and standardized trials are needed to determine optimal strains, dosing, and safety in immunocompromised leukemia patients.^19^^,^^103^

### 4.2 Fecal microbiota transplantation

FMT has demonstrated efficacy in restoring the diversity of gut microbiota in patients undergoing aggressive chemotherapy and antibiotic treatments for AML. This reintroduction of beneficial anaerobes such as *Faecalibacterium* and *Blautia* helps restore SCFA production, reinforce epithelial barrier integrity, and downregulate pro-inflammatory cytokines. This restoration can alleviate dysbiosis, a condition often worsened by cancer therapies, thereby potentially minimizing complications associated with treatment.^62^^,^^79^

Accumulating evidence suggests that FMT may enhance the efficacy of immunotherapy by modulating gut-microbiome composition and improving antitumor immune responses.^62^ FMT may also mitigate complications following chemotherapy and stem-cell transplantation by reducing multidrug-resistant bacteria and promoting overall gut health, critical for immunocompromised patients.^91^ Recent studies indicate that FMT is generally safe for AML patients, with no significant adverse events directly attributed to the procedure, supporting continued investigation of its therapeutic potential.^62^ Although FMT clearly alters gut-microbiota composition, the mechanisms underlying its antitumor effects remain incompletely defined. Current studies suggest that FMT can enhance immune responses, regulate inflammation, and influence tumor microenvironments, yet the microbial species or metabolites responsible for these effects are still uncertain. This uncertainty hampers protocol standardization and underscores the need for further research to refine FMT’s therapeutic use in leukemia and other malignancies.^90^^,^^104^

Donor-to-donor variability in gut-microbiota composition poses a major challenge. The most beneficial donor profiles remain unclear, and there is concern that harmful microorganisms could be transferred to immunocompromised recipients.^81^ Despite encouraging safety data, existing trials are small and methodologically inconsistent. Standardized, large-scale studies are needed to establish optimal donor selection, dosing schedules, and long-term microbiome stability in leukemia patients.^65^^,^^79^

### 4.3 Nanoparticles

Nanoparticle-based drug delivery systems have the potential to improve the effectiveness of chemotherapy by increasing the bioavailability of drugs while reducing adverse effects through targeted delivery to cancer cells. Recent studies also suggest that the efficacy and biodistribution of nanoparticles may be influenced by the gut microbiome, as microbial enzymes and metabolites can alter nanoparticle coating stability and drug-release kinetics. There is ongoing apprehension regarding the long-term toxicity of nanoparticles, as they can accumulate in non-target tissues, potentially resulting in unexpected side effects.^19^ The process for developing and obtaining approval for nanoparticle-based treatments can be protracted and complex due to rigorous regulatory standards.^19^ Moreover, most findings remain preclinical, and data on their long-term immunological and microbiome effects in leukemia patients are still limited.

### 4.4 Peptides

Peptide-based therapies may offer a targeted approach, focusing on particular mechanisms involved in leukemia, which could lead to more effective treatment strategies. Some therapeutic peptides mimic or modulate host–microbe signaling molecules, influencing immune pathways such as TLR activation and cytokine regulation, which may indirectly affect the leukemia microenvironment. The ways in which peptides function can be intricate and differ across various leukemia types, complicating the prediction of treatment outcomes.^19^ However, most available data come from early-phase or preclinical studies, and larger, controlled clinical trials are needed to verify their efficacy, safety, and interaction with existing treatments.

## 5 Future directions and challenges

### 5.1 Gaps in current knowledge of microbiome in leukemia

Research on the microbiome's influence in leukemia is still emerging, exposing substantial gaps in our knowledge. Recent investigations have linked dysbiosis of gut microbiota to different types of leukemia, including AML and ALL, but the precise causal mechanisms are not yet well defined. For example, studies suggest that certain microbial groups, such as *Blautia* and *Lactococcus*, may elevate the risk of developing leukemia; however, the biological processes by which these microbes contribute to leukemogenesis remain largely unexplored.^17^^,^^21^ Future work should incorporate functional studies using gnotobiotic leukemia mouse models or organoid systems to dissect causal pathways between specific microbes, metabolites, and leukemogenesis. Moreover, most studies conducted to date are observational or case-control designs, which complicate the establishment of clear causal links due to potential confounders like age, diet, and environmental factors.^17^^,^^19^ Despite advancements in sequencing technologies that enhance our ability to study microbial communities, defining what constitutes a “healthy” microbiome and addressing individual variability continues to pose challenges.^105^ This situation underscores the necessity for more comprehensive longitudinal studies and innovative experimental approaches to clarify how gut microbiota composition influences both leukemia progression and treatment responses. Integrating longitudinal microbiome profiling with chemotherapeutic response data could help identify microbial biomarkers predictive of toxicity or treatment efficacy, enabling microbiome-guided precision therapies.

### 5.2 Potential for personalized medicine approaches to targeting microbiome in leukemia

The exploration of personalized medicine strategies that focus on the microbiome in leukemia is increasingly prominent, reflecting the intricate relationship between gut microbiota and cancer treatments. Recent research has highlighted that the makeup of the gut microbiome can significantly affect both the effectiveness and adverse effects of therapies, including chemotherapy and immunotherapy.^55^ For example, changes in microbiota diversity during chemotherapy have been associated with negative side effects and differing treatment outcomes among patients with leukemia.^16^^,^^41^ Integrating multi-omics approaches, combining metagenomics, metabolomics, and immune profiling, could enable precise identification of microbial signatures that predict chemotherapy tolerance and immunotherapy responsiveness. By implementing personalized microbiome profiling, clinicians may better predict patient-specific treatment responses and adjust therapy accordingly. This tailored approach aims to enhance therapeutic effectiveness while reducing side effects.^106^^,^^107^ Furthermore, interventions such as probiotics, dietary changes, and FMT are showing potential in improving treatment results by reinstating healthy microbial populations and influencing immune responses.^19^^,^^108^ As studies continue to unveil how gut microbiota impacts leukemia progression and treatment efficacy, integrating microbiome insights into clinical practice may transform the management of this challenging disease.^18^ Such integration will also require developing standardized microbial reference panels and validated clinical pipelines to ensure reproducibility and regulatory compliance in future microbiome-guided leukemia care.

### 5.3 Challenges in developing effective combination-targeted therapies

Creating effective combination-targeted therapies in oncology faces numerous significant hurdles. A major challenge is the limited therapeutic range of targeted agents, which often results in overlapping toxicities that complicate treatment plans. This situation is further complicated by both inherent and acquired resistance in cancer cells, which can develop through clonal selection when subjected to targeted treatments. Emerging evidence also suggests that the gut microbiome can modulate the efficacy and toxicity of these targeted agents by influencing drug metabolism, immune activation, and inflammation, underscoring the need to integrate microbiome considerations into combination therapy design. As a result, more intricate combination strategies are required to address these resistance mechanisms.^109^ Moreover, the sheer volume of potential drug combinations far surpasses the resources available for clinical trials, presenting a formidable challenge for researchers striving to find the best pairings that enhance treatment effectiveness while reducing side effects.^110^^,^^111^ The diversity of tumors adds another layer of difficulty, as individual patients may exhibit different responses to identical combinations due to their distinct genetic and molecular characteristics.^112^^,^^113^ Additionally, practical considerations such as establishing suitable dosing schedules and managing interactions between drugs further complicate the clinical application of these therapies.^109^ In summary, while combination-targeted therapies show great potential for enhancing cancer treatment outcomes, successfully navigating these complex challenges is crucial for their effective development and implementation in clinical practice. Future research should incorporate microbiome profiling and modeling into early-phase combination trials to identify microbial predictors of treatment response, toxicity, and resistance dynamics.

### 5.4 Future research directions

Future investigations into leukemia treatment are increasingly concentrating on targeted therapies and immunotherapies, which seek to boost treatment effectiveness while reducing adverse effects. Noteworthy progress has been achieved with bispecific T-cell engagers, such as blinatumomab, which have demonstrated potential in enhancing survival for patients with ALL who have achieved remission following chemotherapy.^114^ Future research should also examine how the gut microbiome influences patient response to these immunotherapies, as microbial metabolites and immune-modulatory species have been shown to affect T-cell activation and cytokine balance. Furthermore, menin inhibitors, including revumenib, are under examination for their ability to address AML, especially in cases characterized by specific genetic mutations.^115^^,^^116^ Researchers are also exploring the benefits of combination therapies, which merge new agents with established treatments to tackle resistance and enhance patient outcomes. For example, pairing menin inhibitors with conventional chemotherapy may improve treatment success rates for newly diagnosed AML patients.^117^ Additionally, the ongoing investigation into CAR T-cell therapies is promising, with trials assessing their application as first-line treatments for high-risk ALL patients, aiming to prolong remission periods and decrease relapse occurrences.^118^ Collectively, these research initiatives reflect a transition towards more personalized and effective approaches in leukemia management, highlighting the critical need to understand the molecular mechanisms underlying various leukemia subtypes. Integrating longitudinal microbiome profiling and functional metagenomics into these clinical trials could help identify microbial biomarkers predictive of immunotherapy response or toxicity, enabling microbiome-guided precision leukemia care.

### 5.5 Clinical trials of probiotics in leukemia treatment

Several clinical trials have evaluated probiotic supplementation as an adjunct to leukemia therapy, primarily aimed at mitigating chemotherapy-related gastrointestinal toxicity and maintaining microbiome balance.

Trial 1-Probiotic supplementation in preventing treatment-related diarrhea (NCT01644097): A randomized phase II trial assessing the efficacy of *Lactobacillus plantarum* 299v, *Lactobacillus acidophilus*, and *Bifidobacterium lactis* in preventing chemotherapy-induced diarrhea across various malignancies, including leukemia. The intervention aimed to restore microbial diversity and reduce intestinal inflammation.^74^ Trial 2-Effects of probiotics on chemotherapy-induced gastrointestinal side effects: A pilot study investigating *Lactobacillus rhamnosus* supplementation in pediatric leukemia patients. Findings indicated reduced nausea and diarrhea compared to controls, suggesting improved gut resilience during chemotherapy.^75^^,^^76^ Trial 3-Probiotics for oral mucositis in pediatric leukemia: This study evaluated probiotic gargling in children undergoing chemotherapy for leukemia. Significant improvements were observed in oral mucositis scores, highlighting probiotics' potential to mitigate mucosal inflammation.^77^

These studies collectively suggest that probiotic supplementation may reduce treatment-related side effects by supporting microbial balance and mucosal health. However, most trials are limited by small sample sizes, heterogeneous probiotic formulations, and short follow-up periods. Larger, controlled studies incorporating microbiome profiling are essential to validate these findings and guide clinical use.

## 6 Discussion

### 6.1 Summary of current knowledge on microbiome in leukemia and treatment

Recent studies have highlighted the crucial influence of the gut microbiome on the development and treatment of leukemia. However, current evidence is largely correlative; few studies establish direct causality between dysbiosis and leukemogenesis, as most data derive from observational or preclinical models. Alterations in the composition of gut bacteria have been associated with various forms of leukemia, particularly ALL and AML. Research indicates that treatments such as chemotherapy and antibiotics can disrupt the diversity of gut microbiota, a condition that can worsen treatment-related complications and impact patient outcomes.^16^, ^17^, ^18^ For example, a notable decrease in microbial diversity has been documented in ALL patients undergoing induction chemotherapy, underscoring the treatment's effect on gut health.^18^ Additionally, certain microbial groups have been identified as potential risk factors for leukemia; specifically, genera like *Blautia* and *Lactococcus* are linked to a heightened risk for ALL.^17^^,^^118^^,^^119^ To counteract the adverse effects of treatments and improve overall patient outcomes, therapeutic approaches involving probiotics, prebiotics, and FMT are currently under investigation. As research advances, a deeper understanding of the complex relationship between the microbiome and leukemia could pave the way for innovative prevention and treatment strategies. Future research should clarify whether specific microbial metabolites or immune-modulatory pathways, such as short-chain-fatty-acid signaling and T-cell regulation, mediate these effects, thereby enabling targeted microbiome-based adjuvant therapies alongside chemotherapy or immunotherapy.

### 6.2 Importance of microbiome in leukemia research and clinical practice

The role of the microbiome in leukemia research and clinical practice is gaining recognition as a crucial field of investigation, especially concerning its influence on disease initiation, treatment effectiveness, and patient prognoses. Recent studies have revealed that changes in gut microbiota can affect leukemia development, with certain microbial groups associated with a heightened risk of conditions like ALL and AML.^17^^,^^120^ For example, research indicates that mice with genetic predispositions display unique gut microbiomes that can be altered by antibiotic treatments, resulting in leukemia even without infections.^120^ These findings underscore that microbial composition can act as both a modifier and mediator of leukemogenesis, suggesting that host–microbe interactions may influence oncogenic signaling and immune homeostasis beyond infection-related pathways. Additionally, the gut microbiome appears to play a role in affecting both the efficacy and side effects of chemotherapy, implying that maintaining a healthy microbiota could improve treatment results and minimize adverse reactions.^16^^,^^18^^,^^121^ This suggests the possibility of microbiome-focused strategies, such as probiotics or dietary changes, to enhance treatment practices and patient management in leukemia cases.^19^ As investigations continue, a deeper understanding of the complex interactions between gut microbiota and leukemia may lead to new prevention and treatment methods tailored to individual patients. Integrating microbiome profiling into clinical decision-making could enable prediction of treatment response or toxicity and guide the use of adjunct microbiota-modulating interventions such as probiotics or fecal microbiota transplantation.

### 6.3 Need for further research to fully understand the role of microbiome in leukemia and to develop effective combination-targeted therapies

Future investigations should prioritize identifying microbial biomarkers that can predict treatment responses and potential side effects. However, current studies are limited by small sample sizes, inconsistent methodologies, and lack of standardized microbiome profiling, which hinders the identification of reproducible microbial biomarkers. Incorporating microbiome assessments into leukemia management would advance personalized medicine, allowing for tailored therapeutic approaches based on individual microbial profiles.^107^^,^^122^^,^^123^ A deeper understanding of these interactions could facilitate the design of rational combination therapies that exploit microbiome-host crosstalk to enhance efficacy and reduce toxicity. Emerging strategies, such as microbiome-targeted probiotics, engineered bacterial therapies, and precision microbiome modulation, are being explored to enhance immune function and improve leukemia treatment outcomes. Collaborative, multi-omics clinical trials integrating genomics, metabolomics, and microbiome data are needed to elucidate causal pathways and define safe, effective microbiome-based combination therapies.

## 7 Conclusions

The accumulating evidence underscores the gut microbiome as a pivotal modulator of leukemia pathogenesis and treatment response, linking host immunity, metabolism, and therapeutic efficacy. While further investigation is essential to determine the most effective microbial strains and dosages, existing studies indicate that probiotics may assist in enhancing outcomes for leukemia patients by boosting immune function, maintaining gut health, and potentially improving treatment effectiveness. Similarly, FMT shows promise in restoring microbial balance and supporting recovery in leukemia patients, though its optimal clinical use requires further clarification. However, current evidence remains largely correlative and limited by small sample sizes, heterogeneous study designs, and inconsistent microbiome profiling. Larger, well-controlled clinical trials are necessary to establish causality, safety, and standardized protocols for probiotic and FMT applications in hematologic malignancies.

Peptide-based therapies also exhibit significant potential in leukemia management by promoting differentiation in AML and enhancing immune surveillance in CLL. AMPs, such as the PFR peptide, demonstrate synergistic effects with conventional chemotherapy through unique necrotic and immunomodulatory mechanisms. These emerging therapies reflect a broader trend toward targeted, microbiome-informed interventions that seek not only to suppress leukemic proliferation but also to strengthen host defenses.

Nanoparticle-based systems further advance this paradigm by improving drug delivery precision, overcoming resistance, and minimizing systemic toxicity. Yet, their long-term biocompatibility and effects on the gut microbiome warrant continued evaluation. Moving forward, multidisciplinary approaches that integrate microbiome profiling, molecular genomics, and immunotherapy research will be essential to realize the potential of these innovative modalities. Such integration will accelerate the development of precision leukemia therapies, where microbiota-targeted interventions, peptide-based agents, and nanotechnology converge to enhance efficacy, reduce toxicity, and improve survival outcomes for patients across leukemia subtypes.

## CRediT authorship contribution statement

**Saba Jalalifar:** Writing – review & editing, Writing – original draft. **Bahar Bajelan:** Writing – original draft. **Reihane Mohammadi:** Visualization. **Roya Ghafoury:** Supervision, Validation, Writing – review & editing. **Zahra Kalhori:** Writing – review & editing, Writing – original draft, Conceptualization. **Kamran Pooshang-Bagheri:** Writing – review & editing, Supervision, Project administration, Conceptualization. **Reza Nekouian:** Supervision, Project administration. **Mohammad Faranoush:** Supervision, Project administration.

## Informed consent

Not applicable.

## Organ donation

Not applicable.

## Ethical statement

Ethics approval was waived for this study because no patients' data were reported.

## Data availability statement

Data sharing is not applicable to this article as no new data was created or analyzed.

## Animal treatment

Not applicable.

## Generative AI

During the preparation of this work, the authors used ChatGPT to assist with language refinement, grammar correction, and improvement of clarity and readability. After using this tool, the authors carefully reviewed, revised, and edited all generated content and take full responsibility for the integrity, accuracy, and originality of the manuscript.

## Funding

This research did not receive any specific grant from funding agencies in the public, commercial, or not-for-profit sectors.

## Declaration of competing interest

All authors declare that they have no conflict of interest.
